# The impact of direct vertebral rotation (DVR) on radiographic outcome in surgical correction of idiopathic scoliosis

**DOI:** 10.1007/s00402-017-2700-4

**Published:** 2017-04-24

**Authors:** Wiktor Urbanski, Michal J. Wolanczyk, Wojciech Jurasz, Miroslaw Kulej, Piotr Morasiewicz, Szymon Lukasz Dragan, Marek Sasiadek, Szymon Feliks Dragan

**Affiliations:** 1 Department of Orthopaedics and Traumatology, Wroclaw Medical University, ul. Borowska 213, 50-556 Wrocław, Poland; 2 Department of General and Interventional Radiology and Neuroradiology, University Hospital Wroclaw, Wrocław, Poland

**Keywords:** Idiopathic scoliosis, Spinal deformity correction, Apical rotation, Direct vertebral rotation

## Abstract

**Introduction:**

Recent developments of spinal instruments allow to address nearly all components of idiopathic scoliosis. Direct vertebral rotation (DVR) maneuver was introduced to correct apical axial vertebral rotation. It is however still not well established how efficiently DVR affects results of scoliosis correction. The object of the study was to evaluate en bloc apical vertebral rotation (DVR) and its impact on coronal and sagittal correction of the spine in patients undergoing surgical scoliosis treatment.

**Materials and methods:**

Thirty-six consecutive patients who underwent posterior spinal fusion with pedicle screws only constructs for idiopathic scoliosis. Fifteen patients (20 curves) were corrected by rod derotation only and 21 patients (26 curves) had both rod derotation and DVR. Curve measurements were performed on x-rays obtained before and postoperatively—coronal curves, kyphosis (T2–T12, T5–T12). Spine flexibility was assessed on prone bending x-rays. Apical axial rotation was determined on CT scans obtained intraoperatively and postoperatively. Rotation angle (RAsag) was measured according to Aaro and Dahlborn.

**Results:**

We observed reduction of RAsag in all patients; however, in DVR group, decrease was greater, by 31.8% comparing to non-DVR group, by 8.6% (*p* = 0.0003). Mean coronal correction in DVR group was 68.8% and in rod derotation group without DVR 55% (*p* = 0.002). No significant correlation was found between degree of derotation obtained and coronal correction. In DVR group T2–T12 kyphosis has increased in 28 (65%) patients whereas in non-DVR group in 31 (69%) cases. Mean value of T2–T12 kyphosis growth was 16.7% in DVR and 22.1% in non-DVR group. These differences however did not occur statistically significant.

**Conclusions:**

Direct vertebral rotation (DVR) maneuver reduces significantly apical rotation of the spine, enhances ability of coronal correction, and it does not reduce thoracic kyphosis.

## Introduction

Idiopathic scoliosis is a three-dimensional deformity of the spine. Modern solutions of spinal instrumentations allow addressing all components of the deformity—sagittal, coronal, and axial. Vertebral apical rotation in scoliosis contributes to the development of rib hump—which is considered as a very significant impairment for the patient [1, 2]. Moreover, from patient’s perspective, chest deformity itself may be a reason for surgery. Traditionally to dispose of the rib hump, thoracoplasty has been performed, although this procedure might be related to serious complications and comorbidities such as increased blood loss, persistent pain, pneumothorax, negative impact on pulmonary function, and extended time of surgery [3, 4].

The object of surgical treatment of deformed spine is to prevent curve progression, attain maximal deformity correction, and to obtain balanced spine with proper sagittal alignment and minimal spine fusion. Direct vertebral rotation (DVR) was developed with the expectation to complement correction of twisted spine, to overcome complications in rib hump reduction associated with thoracoplasty, as well as to reduce fusion extension and optimize correction in coronal and sagittal planes [2, 5–8]. DVR maneuvers consist of derotation of apex vertebrae (levels of greatest rotation and translation due to spinal deformity) and correction of the axial spinal deformity. The technique is usually done after basic corrective maneuvers such as translation, rod derotation, or in situ bending.

However, there is a still lack of evidence whether DVR contributes to better clinical outcome [9]. Yet it has not been well established how correction of axial deformity (vertebral rotation) affects coronal and sagittal spinal alignment. Some surgeons suggest that DVR has a hypokyphotic effect on thoracic kyphosis, increases the risk of pull out of the screws, and prolongs the surgical time without other clear benefits [9–13].

The purpose of presented study was to evaluate the effect of *en bloc* direct vertebral rotation (DVR) maneuver on true vertebral rotation, coronal, and sagittal alignment as assessed by imaging studies in patients treated surgically for idiopathic scoliosis.

## Materials and methods

Thirty-six consecutive patients (5 males, 31 females) after correction of progressive adolescent and neglected adult idiopathic scoliosis were included in the analysis. Two independent radiologists (MW, MS) conducted all radiographic measurements on plain x-rays and CT scans obtained with O-arm (Medtronic) postoperatively. Measurements were performed on standing, long film x-rays, obtained before and shortly after surgery. Cobb angles in coronal plane curves and sagittal profile (kyphosis T2–T12, T5–T12, and lordosis L1–S1), as well as the extent of their correction following surgery, were described. On prone bending X-rays curve flexibility was assessed. Curves that did correct 40% or more were considered as flexible whereas stiff curves were correctable less than 40%.

**Fig. 1:**
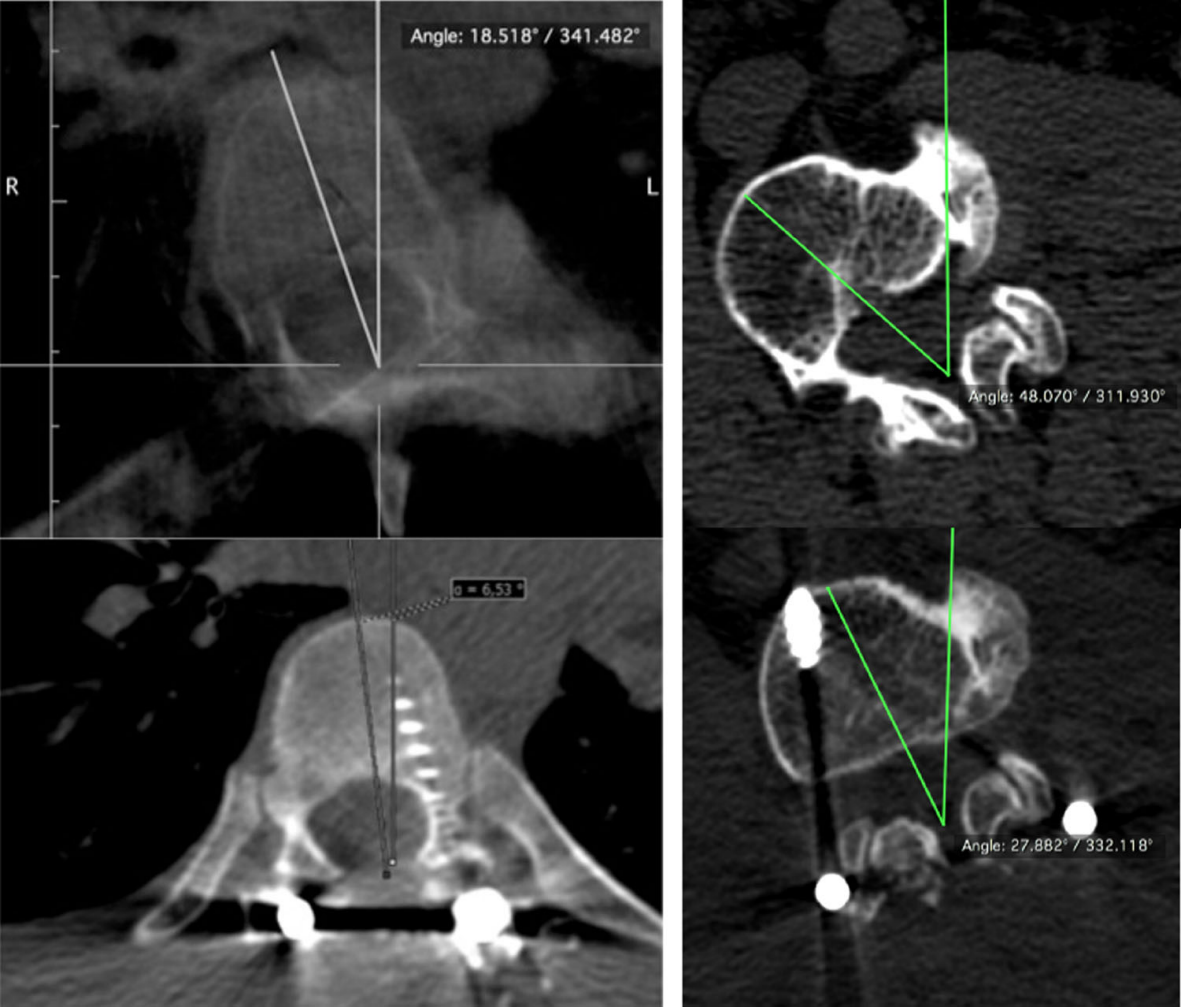
Axial scans. RAsag before (*upper*) and after surgery (*below*), DVR group

Axial apical vertebral rotation (AVR) was determined on CT scans according to the method described by Aaro and Dahlborn [14, 15]. The rotation angle (RAsag) was measured in relation to neutral vertebrae. The assessment of RAsag was made pre- and postoperatively (Fig. 1).

**Fig. 2:**
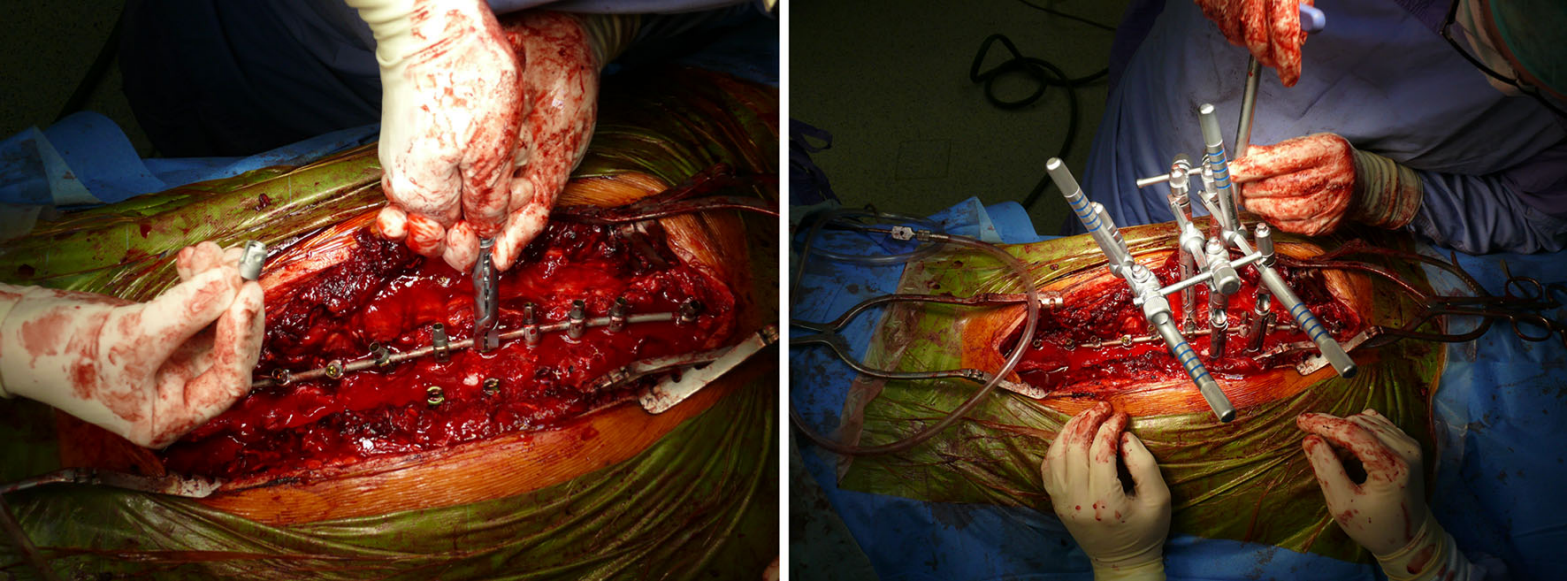
**a** Mounting of VCM device for DVR. On each screw at the curve apex, implant holder is attached. **b** Implant holders linked to each other with the set of connectors creating stiff construct

**Fig. 3:**
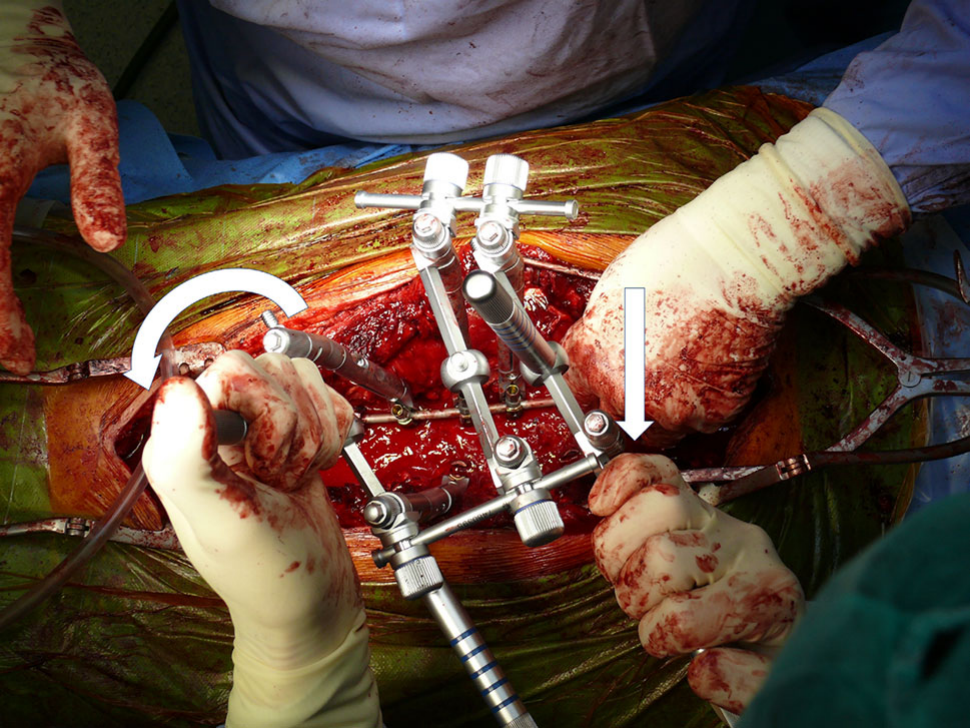
The maneuver of derotation; the entire construct in *en bloc* manner rotates horizontally the scoliosis apex. *White arrows* show the direction of applied force on VCM

All patients underwent posterior spinal fusion only with all screw constructs and 70–90% screw density. Correction and instrumented fusions were done with 5.5 titanium instrumentation (Legacy/Solera, Medtronic). Prior to corrective maneuvers (rod derotation by 90° of the concave rod, mild under-contouring of the convex one), all patients underwent apical posterior release: Ponte osteotomy, flavectomy. Randomly chosen patients received apical direct vertebral rotations (DVR), performed with Vertebral Column Manipulator device (VCM, Medtronic) following the description of Lenke et al [16]. Patients with pronounced rib/loin hump hence significant apical vertebral rotation received DVR procedure, however no particular criteria of inclusion were applied. Immediately after curve correction by 90° rod derotation, VCM was mounted over apex screws, the level above and below (3 levels) (Fig. 2). In order to get an efficient axial correction/derotation, either monoaxial or uniplanar screws were used at the levels undergoing DVR. Once VCM construct was assembled, forceful derotation was done in *en bloc* manner—three levels connected in one stiff construct, resulting in derotation force applied evenly to the whole apex (Fig. 3).

Fourteen (20 curves) patients underwent correction by rod derotation only and 22 (26 curves) underwent rod derotation and direct vertebral rotation (DVR). The data were analyzed separately for DVR and non-DVR group in adults and adolescents, thoracic and lumbar spine, and in rigid and flexible curves. Kyphosis at T2–T12 and T5–T12 levels were assessed.

Statistical assessment and significance of differences in analyzed groups were performed with *t* student and Mann–Whitney tests. Mann–Whitney *U* test was used when both groups had less than 20 measurements each, while Mann–Whitney *Z* test when one of the group had ≥20 measurements. In the case of non-compliance homogeneity of variance and/or the presence of normal distribution (*p* > 0.05), we could not use the student t-test and selected the Mann–Whitney test. The correlation coefficient was used to determine the relationship between two properties. A *p* value less than 0.05 was statistically significant.

## Results

Preoperative curves magnitudes, their flexibility, AVR, and age distribution were similar in DVR and non-DVR groups (Table 1). In DVR group, major structural thoracic curves dominated over double structural thoracolumbar ones (Lenke 1 and 2–72.7%, Lenke 3, 4, 6–18.2%), while in non-DVR group, double major thoracolumbar and major thoracic curves were evenly distributed (Lenke 1 and 2–50%, Lenke 3, 4, 6–50%).

**Table 1:** Preoperative characteristics of patients

Preoperative parameter	DVR (22 patients)	Non-DVR (14 patients)	*P* value
Age at surgery	18 years (11–30 years) ± 4.53	21 (13–43 years) ± 7.21	0.104
Adults 21–43 years	7 curves (7 patients) Mean age 21.9 years ± 4.5	12 curves (9 patients) mean age 24.8 years ± 6.98	0.347
Adolescents 11–17 years	19 curves (15 patients) Mean age 14.9 years ± 1.58	8 curves (5 patients) mean age 15.6 years ± 1.49	0.464
Sex (male/female)	2/20	4/10	0.133
Flexibility	41.4 ± 12.07	33.8 ± 18.02	0.127
RAsag (AVR)	20.16 ± 5.93	23.36 ± 5.81	0.128
Cobb angle	58 (40–84) ± 11.65	65.5 (45–95) ± 13.08	0.096
Kyphosis T2–T12	34.41 ± 11.26	37.15 ± 17.21	0.626
Kyphosis T5–T12	27.68 ± 11.07	26.23 ± 14.45	0.766

±Standard deviation

**Fig. 4:**
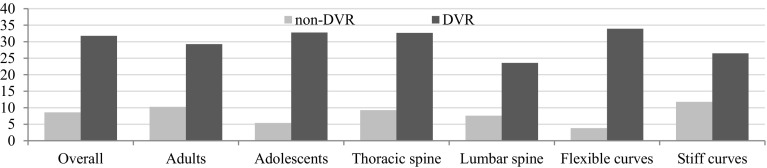
Rotation angle (RAsag) change

*Axial vertebral rotation* Regardless of analyzed features (age, curve flexibility, the region of the spine), DVR maneuver produced greater RAsag reduction than rod derotation only (Fig. 4); however, statistical significance was not reached for lumbar spine and stiff curves (Table 2). The impact of DVR on RAsag alteration was more pronounced in adolescents than adults, in flexible than stiff curves, and in thoracic than lumbar spine (Fig. 4); nonetheless, the analysis did not confirm any statistical significance.

**Table 2:** Postoperative change of parameter value

Parameter %	Non-DVR	DVR	*P* value
RAsag reduction			
Overall	8.6 ± 17.58	31.8 ± 17.50	*0.0003*
Adults	10.3 ± 20.52	29.3 ± 12.57	*0.0498*
Adolescents	5.4 ± 8.31	32.8 ± 19.03	*0.0007*
Thoracic spine	9.3 ± 17.54	32.7 ± 17.71	*0.0048*
Lumbar spine	7.6 ± 17.60	23.6 ± 13.96	0.1642
Flexible curves	3.83 ± 20.37	33.93 ± 16.72	*0.0192*
Stiff curves	11.8 ± 14.58	26.5 ± 18.26	0.165
Coronal correction			
Overall	55.08 ± 15.63	68.8 ± 13.41	*0.002*
Adults	50.0 ± 12.06	66.1 ± 11.80	*0.0212*
Adolescents	62.1 ± 17.21	69.8 ± 13.83	0.3117
Stiff	55.18 ± 17.63	60.98 ± 17.96	0.5288
Flexible	55.0 ± 12.37	71.7 ± 9.82	*0.0094*
Lumbar	59.71 ± 19.52	72.68 ± 16.48	0.2573
Thoracic	49.43 ± 15.44	67.65 ± 12.11	*0.0022*
Kyphosis increase T2–T12			
Overall	22.1 ± 42.61	16.7 ± 42.87	0.824
Adults	13.4 ± 27.71	38.4 ± 57.9	0.3618
Adolescents	35.9 ± 56.41	6.6 ± 28.43	0.3659
Flexible curves	18.5 ± 9.14	20.3 ± 47.46	0.8969
Stiff curves	23.16 ± 48.27	4.56 ± 15.36	0.3172
Kyphosis increase T5–T12			
Overall	35.6 ± 47.72	24.9 ± 45.69	0.645
Adults	22.2 ± 44.41	48.6 ± 63.20	0.4192
Adolescents	54.5 ± 45.76	13.8 ± 28.50	0.1536
Flexible curves	15.4 ± 19.86	30.9 ± 49.93	0.4412
Stiff curves	42.3 ± 52.18	4.5 ± 13.09	0.0822

±Standard deviation. Italicized *p* < 0.05

**Fig. 5:**
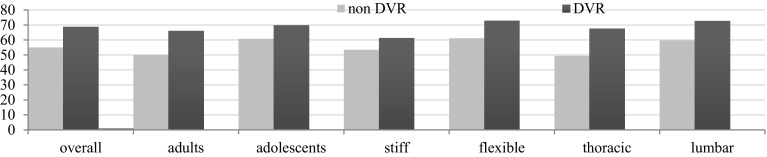
Mean percentage of coronal correction

*Coronal* Mean general correction of coronal spinal deformity was 63.01% ± 15.91 in the whole group. DVR maneuver provided better coronal correction than simple rod derotation without DVR in general (mean 68.8 and 55%, respectively, *p* = 0.002, Table 2) as well as in regards of analyzed parameter (Fig. 5).

The greater impact of DVR on coronal correction was observed for flexible than stiff curves (16.7 and 5.8% of improvement, respectively), in adults than in adolescents (16.1 and 7.7%), and in thoracic than lumbar spine (by 18.22 and 12.97%) (Table 2).

However, no significant correlation was found between degree of derotation obtained and coronal correction.

*Kyphosis* In DVR group, T2–T12 kyphosis has increased in 28 (65%) patients, whereas in non-DVR group in 31 (69%) cases. Mean value of T2–T12 kyphosis growth was 16.7% in DVR and 22.1% in non-DVR group. For T5–T12 in DVR group, kyphosis has increased in 30 (80%) patients and in non-DVR group 32 (78%) with mean value increase by 24.9 and 35.6%, respectively. These differences, however, did not occur statistically significant (Table 2). DVR improved considerably thoracic kyphosis in adults and flexible curves, and in adolescents and stiff curves on the contrary, we have noticed only minor kyphosis increase in DVR group and the considerable increase in non-DVR (Table 2). None of these observations were proven significant statistically, however.

## Discussion

DVR has become a popular technique complementing surgical scoliosis treatment. Nevertheless, questions remain unanswered regarding its supportive role, safety, and impact on the general outcome. Most of the previously published studies describing the role of the DVR maneuver do not focus on the detailed radiological outcome of the technique. Presented paper is the analysis of 36 consecutive patients after IS correction and PSF. Series of radiographic measurements has been made to determine whether DVR affects scoliosis correction and under what circumstances (rigid/flexible curves, adults/adolescents, thoracic/lumbar spine) it may provide the best correction of spinal deformity.

The value of this report is that all the patients had CT scans performed pre- and postoperatively what provided information regarding alteration of true vertebral rotation during operation. 3D imaging allowed to assess relations between axial rotation and coronal and sagittal profile correction. Poor homogeneity of DVR and non-DVR groups in terms of curve pattern (Lenke classification) and small numbers of patients in evaluated groups may be considered as the study limitation.

According to biomechanical studies, axial rotation in scoliosis is an integral part of deformity and it contributes to coronal and sagittal components. This phenomenon is known as the “coupling” of rotation and translation between anatomic axes [17–19]. Based on coupled motions of the spine, 3-dimensional correction with DVR appears to be an obvious component of scoliosis correction and should deliver an overall better result.

In the presented study, DVR provided the clear improvement of coronal correction by nearly 14% in the whole series, especially effective in adults (by 16%) and in flexible curves (by nearly 17%). Several authors reported similar results. Di Silvestre et al., Kadoury et al., Samandi et al. presented 8–10% better correction in coronal plane caused by DVR application [5–7]. In Lee’s and Suk’s papers, the differences were even more significant in favor of DVR. [2, 8]. On the other hand, Matilla et al. have not found any difference in coronal correction between DVR and standard rod rotation [20]. Additionally, in our study, an attempt was made to evaluate a possible correlation between the amount of derotation and coronal correction; however, no such correlation was established.

Most patients with adolescent idiopathic scoliosis (AIS) are primary hypokyphotic in the thoracic region and the application of pedicle screws although improves correction in coronal plane seems to have a detrimental effect on sagittal profile—decrease of thoracic kyphosis [21, 22]. According to Dickson’s theory of AIS development [23, 24] supported later by Guo [25], anterior column overgrowth leads to lordotization and concomitant lateral “buckling” of the spine. Basing on this theory, axial correction of AIS spine (DVR) shall inevitably result in further decrease of thoracic kyphosis [26]. In the 3D simulation study [10], kyphosis was reduced after complete correction of the coronal and rotational deformity, but it was maintained after the coronal-only correction (2.7° vs. 15°). Although this simulation was established to correct axial rotation completely (to 0°), the situation not existing in clinical practice, there are clinical reports from DiSilvestre [5] and Mladenov [11] supporting this view—they both observed lower kyphosis results in DVR group in comparison to standard rod rotation alone. On the other hand, there are contradictory observations from Hwang et al. who reported the decrease in postoperative kyphosis in the whole series of patients but did not find any worsening of the sagittal profile in DVR group [27]. Furthermore, Mattila et al. suggested that DVR besides significant effect on spinal column derotation might help prevent kyphosis flattening [20]. Similarly, Lee and Suk presented the average thoracic sagittal correction kyphosis of 7° in the DVR group and only of 5° in the standard rod rotation group [8].

In the presented study, 65% of DVR patients had improvement of kyphosis with mean increase smaller (by 16.7%) than in non-DVR patients (by 22.1%), but we did not notice the clear lordotic effect. DVR maneuvers in all cases were performed en bloc and according to Hwang, this technique has a lesser lordotic effect than segmental or other derotation methods. The evidence is not strong—only one study with relatively small numbers represented in the en bloc group [27]; however, data presented in our study support Hwang’s observations.

Since thoracic kyphosis in scoliosis is mainly altered in the mid-thoracic region (apex of scoliosis) in order to describe fully the influence of instrumentation and corrective maneuvers on kyphosis, measurements were performed between T5–T12 and T2–T12 levels. Authors noticed the more evident increase of T5–T12 than T2–T12 kyphosis what suggests true improvement of the most lordotic region and sagittal profile alteration.

We have decided to assess axial apical rotation on CT scans since it is the only method to measure it precisely [28, 29]. Reported efficacy of vertebral derotation varies widely, yet direct vertebral rotation showed the significantly better reduction of apical rotation over other methods [5, 8, 30–32]. In our series, axial rotation changed significantly by 31.8% in DVR group comparing to 8.6% in non-DVR. We have noticed benefits of derotation maneuver on the coronal and sagittal plane, but unfortunately, we did not find any correlation between the amount of derotation achieved and coronal or sagittal correction.

This paper contains only radiographic data and it may be considered as a limitation since the radiographic result is mainly of interest to surgeons but of less importance to patients [33]. Still, there is no clear evidence that application of DVR benefits in terms of clinical outcome and patient’s self-assessment [9]; thus, analysis of the clinical effect of DVR is required.

Obtained data and our previous experience suggest that DVR technique with the apical posterior release and use of highly rigid rods enhance the radiological result of scoliosis corrective surgery, particularly in stiff curves and adults. Moreover, en bloc DVR maneuver does not reduce thoracic kyphosis.

## Conclusions

- Direct vertebral rotation (DVR) maneuver enhances ability of coronal correction.
- En bloc DVR does not reduce thoracic kyphosis; in fact, it increases it, but not as well as rod rotation only.
- DVR is more efficient in thoracic than lumbar area—better RAsag reduction and coronal plane correction.
- No correlation was found between amount of axial derotation and result of coronal and sagittal plane correction.
